# Description of *Caenorhabditis sinica* sp. n. (Nematoda: Rhabditidae), a Nematode Species Used in Comparative Biology for *C. elegans*

**DOI:** 10.1371/journal.pone.0110957

**Published:** 2014-11-06

**Authors:** Ren-E Huang, Xiaoliang Ren, Yifei Qiu, Zhongying Zhao

**Affiliations:** 1 School of Life Sciences, Tsinghua University, Beijing, China; 2 Department of Biology, Faculty of Science, Hong Kong Baptist University, Hong Kong, China; Centre National de la Recherche Scientique & University of Nice Sophia-Antipolis, France

## Abstract

We re-isolated in China a relative of the nematode model *Caenorhabditis elegans* that was previously referred to informally as *C.* sp. 5. In spite of its importance for comparative biology, *C.* sp. 5 has remained morphologically uncharacterized. Therefore, we now provide detailed description of morphology and anatomy, assigning the name of *Caenorhabditis sinica* sp. n. to this nematode that is found frequently in China. *C. sinica* sp. n. belongs to the *Elegans* group in the genus *Caenorhabditis*, being phylogenetically close to *C. briggsae* although differing in reproductive mode. The gonochoristic *C. sinica* sp. n. displays two significantly larger distal parts of uteri filled with sperms in the female/hermaphroditic gonad than does the androdioecious *C. briggsae*. The new species can be differentiated morphologically from all known *Caenorhabditis* species within the *Elegans* group by presenting a uniquely shaped, three-pointed hook structure on the male precloacal lip. The lateral field of *C. sinica* sp. n. is marked by three ridges that are flanked by two additional incisures, sometimes appearing as five ridges in total. This study ends the prolonged period of the ‘undescribed’ anonymity for *C. sinica* sp. n. since its discovery and use in comparative biological research. Significant and crossing-direction dependent hybrid incompatibilities in F1 and F2 crossing progeny make *C. sinica* sp. n. an excellent model for studies of population and speciation genetics. The abundance of nematode species lacking detailed taxonomic characterization deserves renewed attention to address the species description gap for this important yet morphologically ‘difficult’ group of animals.

## Introduction

In recent years, a new *Caenorhabditis* nematode informally referred to as *C.* sp. 5 has been discovered, and used in comparative developmental and evolutionary studies for the genetic model organism *C. elegans* as well as its close relatives [1]–[11]. A draft genome of this species was also assembled [12]. Unlike *C. elegans* and *C. briggsae* being androdioecious with selfing hermaphrodites and facultative males, *C.* sp. 5 is a gonochoristic obligate female/male out-crosser, which displays an ancestral mode of reproduction for the genus *Caenorhabditis*, though this species is closely related to *C. briggsae* phylogenetically [13]. *C.* sp. 5 has been reported to be commonly found in eastern Asia and widely distributed throughout China [14], [15]. The first strains of this species are isolates JU727 and SB378, which were found in the soil under a tree in Chengyang, 20 km North of Sanjiang, Guangxi Province, China and in the soil from a flowerbed in a park in Guangzhou, Guangdong Province, China, respectively [1]. Interestingly, although *C.* sp. 5 exhibits molecular hyperdiversity, this species is apparently restricted to East Asia within a small geographic range [13]–[15]. Despite the numerous interests on this species with its importance to evolutionary genetics, *C.* sp. 5 unfortunately remains morphologically undescribed and formally unnamed yet, hindering its use in molecular and population genetic studies.

During our sampling of nematode species within the Guangdong Province, China, *C*. sp. 5 was re-isolated frequently. According to Kiontke et al. [13], the internal transcribed spacer 2 (ITS2), the nucleotide sequence of the intergenic region between 5.8S and 28S (LSU) rRNA genes, provides a reliable barcode for initial identification of *Caenorhabditis* spp. Using ITS2 barcode sequence combined with morphological data, we identified five (ZZY0401, ZZY0402, ZZY0403, ZZY0413, ZZY0414) out of a total of 17 isolates recovered in this survey as *C*. sp. 5. Crossing tests between our isolates and *C.* sp. 5 reference strain JU727 as well as its another isolate JU1201 confirmed the previous reports that this species exhibits very high population polymorphism, genetic variation and some degree of genetic isolation between populations [14], [15]. Given its relevance to evolutionary and comparative biology, this anonymous nematode species is here morphologically characterized and illustrated in detail and described as *Caenorhabditis sinica* sp. n.

## Materials and Methods

### Isolation and culturing of *Caenorhabditis sinica* sp. n

*Caenorhabditis sinica* sp. n. was described and illustrated using a wild isolate ZZY0401, which was recovered from a rotten fruit in Luofu Mountain of Huizhou City, Guangdong Province, China (our sampling activities were conducted using rotten plant tissues in soil, e.g. rotten fruits or leaves, so no specific permissions were required; and nematodes extracted from these rotten tissues are free-living soil worms, which are no harm to humans, animals, plants and our environment). Individuals were picked out and multiplied on NGM agar plate seeded with bacteria *Escherichia coli* OP50 at room temperature.

### Microscopy and morphological description

Measurements [16], drawings and descriptions were made with the adult animals recovered from the culture plate. Differential interference contrast (DIC) images were taken with an Eclipse Ti Inverted Microscope (Nikon). For scanning electron microscopy (SEM), a modification of Wergin's method [17] was used: living adults were heat-relaxed, fixed in 2.5% glutaraldehyde in M9 buffer, dehydrated through a graded ethanol series, critical-point-dried and sputter-coated with gold-palladium. Permanent slides were made through fixation in 3% formaldehyde followed by gradual dehydration of the nematodes to glycerin using the method of Ryss [18].

### Crossing and genotyping

To determine conspecificity of the reported *C. sinica* strain JU1201 which recovered from a small fruit in the garden of Suzhou City, Jiangsu Province, China [14] and our own isolates genetically, we crossed the strain with our five isolates (ZZY0401, ZZY0402, ZZY0403, ZZY0413 and ZZY0414) in both directions. More specifically, five young adult males of JU1201 and five L4 females of our own isolate were mated overnight on a single plate at room temperature and transferred to a fresh plate to lay egg for another five hours. The parental animals were picked off and the eggs laid on the new plate were allowed to develop for another 24 hours at 25°C before scoring the embryonic lethality, which was defined as the percentage of unhatched embryos out of the total embryos on the plate. The crossings were performed in five replicates. Sex ratio was scored on each plate after another 48 hours. Sex ratio was measured as the number of F1 adult male divided by the total number of adult F1 progeny. The crossing was also performed in opposite direction and the adult male and female progeny were readily identified (data not shown) but not scored for the embryonic lethality and sex ratio.

To further confirm the conspecificity between our five wild isolates and the reference strain for *C. sinica* sp. n., we performed the similar crossings between the standard reference strain JU727 (which was obtained from *Caenorhabditis* Genetics Center) and our five isolates as that with JU1201 but scored embryonic lethality in the F1 hybrid progeny in both directions separately. To determine whether there was any hybrid breakdown of progeny in the subsequent generation, we conducted the crossing between the F1 males and F1 females and scored embryonic lethality in F2 progeny in the similar way as that for the F1 progeny.

For molecular barcoding analysis, genomic DNA used as a PCR template was extracted using PureLink™ Genomic DNA Mini Kit (Invitrogen, USA). Different sets of primers were used in the PCR reactions. Primer sequences for ITS2 were reported previously [13]. Forward and reverse primer sequences used for 18S (SSU) rRNA amplification were SSUG18S [19] and SSU24R [20], RHAB1350F and RHAB1868R [21], as well as SSU24F and SSU18P [19], respectively. Forward and reverse primer sequences for 28S (LSU) D2/D3 rRNA amplification were no. 391 [22] and no. 501 [23]. PCR amplification was performed in a 20 µl reaction system containing 0.1 µl Ex Taq DNA polymerase mix, 1 µl each of 10-µM forward and reverse primers and 2 µl of DNA template. The thermal cycling settings were as follows: denaturation at 95°C for 10 min, followed by 35 cycles of denaturation at 95°C for 45 s, annealing at 55°C for 45 s and extension at 72°C for 2 min. A final extension was performed at 72°C for 10 min. PCR product was purified using KingFisher magnetic particle processor (Thermo Scientific). PCR primers were also the sequencing primer. The sequences were deposited into the GenBank database and compared against those of other strains for *Caenorhabditis sinica* sp. n. available at the GenBank using the BLAST program.

### Nomenclatural acts

The electronic edition of this article conforms to the requirements of the amended International Code of Zoological Nomenclature, and hence the new names contained herein are available under that Code from the electronic edition of this article. This published work and the nomenclatural acts it contains have been registered in ZooBank, the online registration system for the ICZN. The ZooBank LSIDs (Life Science Identifiers) can be resolved and the associated information viewed through any standard web browser by appending the LSID to the prefix “http://zoobank.org/”. The LSID for this publication is: urn:lsid:zoobank.org:pub:888EED23-4C79-4C23-8D04-C5D1EB1441C5. The electronic edition of this work was published in a journal with an ISSN, and has been archived and is available from the following digital repositories: PubMed Central and LOCKSS.

## Results

### *Caenorhabditis sinica* Huang, Ren, Qiu & Zhao sp. n

**Figure 1:**
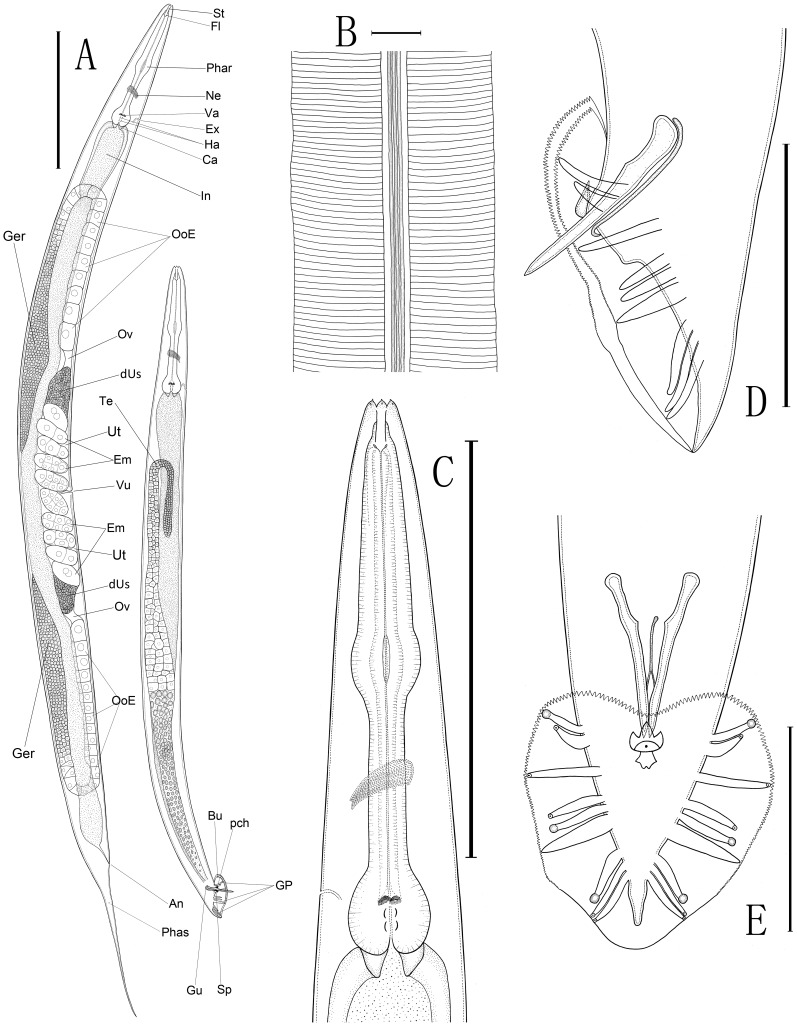
Line drawings of *Caenorhabditis sinica* sp. n. A: Overall anatomy of female (left) and male (right)[St: stoma, Fl: flap, Phar: pharynx, Ne: nerve ring, Va: valvular apparatus, Ha: haustrulum, Ex: excretory pore, Ca: cardia (the pharyngo-intestinal valve), In: intestine, Ger: “germigen” containing a well-developed central rachis surrounded by a layer of germ cells, OoE: elongated oocytes, Ov: oviduct, dUs: the distal part of anterior/posterior uterus filled with sperms, Ut: uterus, Em: embryos carried by the uteri, Vu: vulva, An: anus, Phas: phasmid, Te: testis, Bu: bursa, GP: genital papillae, Gu: gubernaculum, Sp: spicule, pch: precloacal hook]; B: Morphology of lateral field (3 ridges flanked by two additional incisures); C: Anterior region of female; D: Lateral view of male caudal region; E: Ventral view of male caudal region with bursa and genital papillae. (Scale bars: A = 200 µm; B = 10 µm; C = 150 µm; D, E = 50 µm).

**Figure 2:**
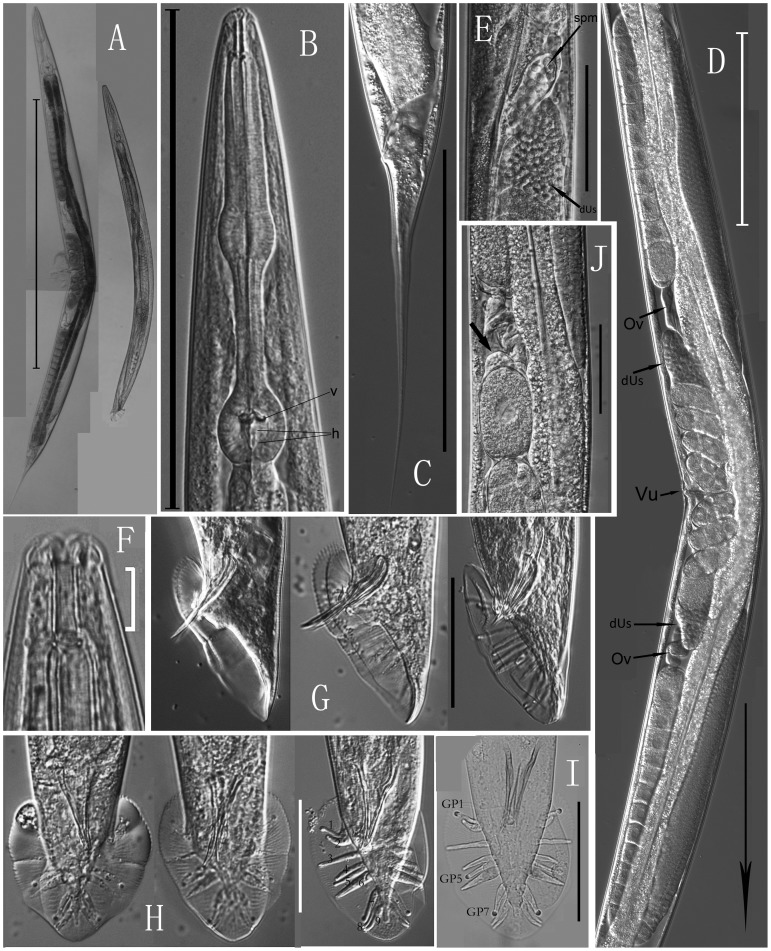
Micrographs of *Caenorhabditis sinica* sp. n. (A–I) A: Entire view of female (left) and male (right); B: Pharynx (v: valvular apparatus; h: haustrulum); C: Female tail; D: Didelphic-amphidelphic female gonad (Ov: oviduct, Vu: vulva, dUs: the distal part of anterior/posterior uterus filled with sperms, arrow shows the posterior direction); E: Spermatheca formed by the oviduct (spm) and the distal part of anterior uterus (dUs); F: Anterior end of female; G: Lateral view of male caudal regions; H, I: Ventral view of male caudal regions showing bursa or its nine pairs of genital papillae (GP1, GP5, GP7: terminated on the dorsal side by a conspicuous sensillum tip). J: The distal part of anterior uterus of a wild *C. briggsae* strain ZZY0405 (indicated by arrow). (Scale bars: A = 800 µm; B, C, D = 200 µm; E = 100 µm; F = 10 µm; G, H, I, J = 50 µm).

**Figure 3:**
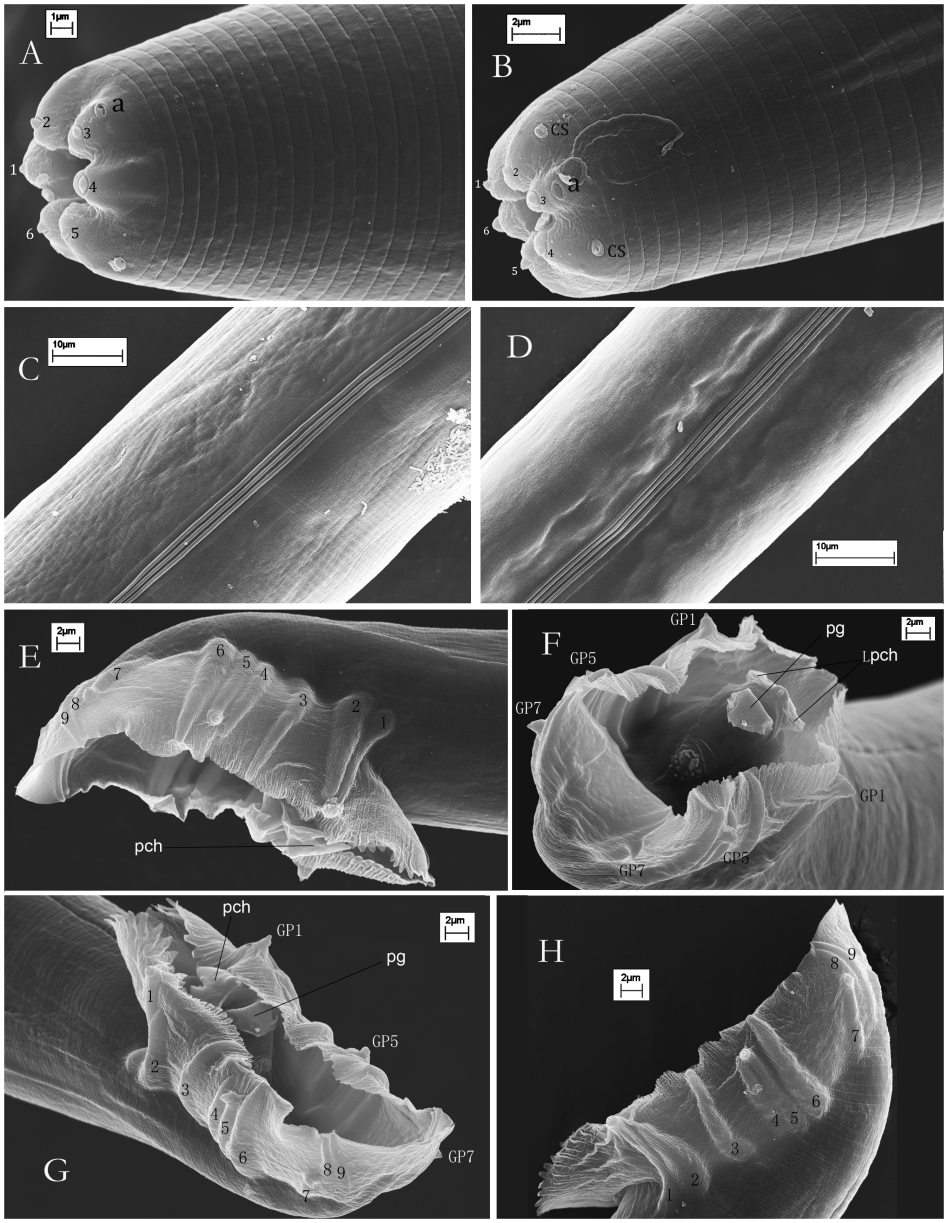
Scanning electron micrographs of *Caenorhabditis sinica* sp. n. A, B: Anterior regions of female (A) and male (1–6: six labial sensilla; a: amphid; CS: cephalic sensilla, which are present only in males); C: Lateral field of female; D: Lateral field of male; E, F, G, H: Male caudal regions enveloped by a closed bursa and its nine pairs of genital papillae (pch: precloacal hook; Lpch: the two lateral points of the precloacal hook; pg: posterior part of gubernaculum; GP1, GP5, GP7: showing their dorsal tips being of typical papilliform sensilla).

urn:lsid:zoobank.org:act:C17880C9-8AC0-448B-A2FB-083FEEED3911 =  *Caenorhabditis* sp. 5 in [1], [13] (Figs 1–3).

### Description

#### Adult

Cuticle surface bears parallel fine transverse striations (Figure 3A & B). Lateral field in the mid-body region consists of three middle ridges plus two lateral incisures (Figure 3C & D). Sometimes a total of five ridges are observed especially for a drying shrinkage. Lip region is continuous with adjoining body contour with six lips closed. Each lip apically bears one external papilliform labial sensillum. Amphidial apertures are conspicuous in SEM, pore-like, situated slightly posterior to the middle of lateral lips (Figure 3A & B). Four additional papilliform cephalic sensilla are present in males only, located near the base of the sub-dorsal/sub-ventral lips (Figure 3B). Anterior part of the stoma (Cheilorhabdion) is weakly cuticularized. Main part of stoma is often slightly funnel-shaped. Metastegostom only slightly bulges into stoma lumen, each sector bearing a visible flap (Figure 2E). Anterior part of pharynx ( =  pro + metacorpus) is slightly longer than posterior section (isthmus + basal bulb). Procorpus muscular, stout, occupies half to two-thirds of corresponding body diam. Metacorpus is muscular, forming swollen median bulb. Isthmus is narrow. Terminal bulb is conspicuous with double haustrulum posterior to valvular apparatus (Figure 2B). The pharyngo-intestinal valves (cardia) are prominent. Nerve ring is around the middle of isthmus. Excretory pore positions are variable, usually around the level of valves in the terminal bulb (ca 15 µm anterior or posterior to this level). Measurements are listed in Table 1.

**Table 1:** Morphometrics of *Caenorhabditis sinica* sp. n. [16]. All measurements are in µm and shown as in the form: mean ± s.d. (range).

Character	14 females	12 males
L	1531.9±215.4	959.81±149.88
	(1102.3–2007.3)	(686.79–1150.18)
a	17.77±1.52	18.44±2.24
	(15.52–20)	(15.75–23)
b	6.78±0.65	5.23±0.69
	(5.3–7.79)	(4.37–6.81)
c	6.5±0.7	19.06±1.78
	(5.09–7.61)	(15.47–21.25)
c′	7.08±0.74	1.73±0.24
	(6.18–8.74)	(1.43–2.29)
V	49.26±0.96	–
	(48–51)	–
Stoma length	19.76±2.18	18.85±0.84
	(16.34–24.27)	(17–20)
Stoma diam.	4.08±0.73	2.89±0.35
	(2.67–5.44)	(2.02–3.36)
Pharynx length	198.04±13.43	172.5±8.14
	(174.06–218.45)	(150–181)
Anterior part of pharynx length	105.75±6.24	91.77±4.54
	(94.66–116.15)	(82.35–96.99)
Posterior part of pharynx length	93.07±8.52	80.73±4.63
	(77.83–106.8)	(67.65–85.95)
Diam. of median bulb	24.95±2.03	19.17±1.53
	(20.08–29.24)	(16.47–22.59)
Diam. of terminal bulb	30.79±2.84	24.01±2.23
	(24.41–35.29)	(19.12–26)
Anterior gonad branch length^1^	808.52±76.77	–
	(618.85–929.61)	–
Posterior gonad branch length^2^	751.9±66.73	–
	(586.07–844.86)	–
The total length of gonad^3^	1560.42±139.12	711.89±92.7
	(1204.92–1774.27)	(531.08–801.48)
Vulva body diam.	81.64±14.36	–
	(55.19–107.45)	–
Vulva to anus distance	533.61±105.94	–
	(355.46–771.67)	–
Tail length	229.71±18.8	49.46±4.35
	(201.46–263.74)	(44–58.08)
Spicule length	–	41.32±2.32
		(38.68–45.98)
Gubernaculum length	–	32.75±2.19
		(29.06–36)
Gubernac. length as % spicule length	–	79.28±3.94
		(72.65–85.65)

1 From vulva to anterior end;

2 from vulva to posterior end;

3 from cloaca to anterior end in the male.

#### Female

Body is almost straight or slightly ventrally arcuate in fixed specimens. Didelphic, amphidelphic gonad is well-developed, stretching more than half the length of the entire body with both ovaries antidromously reflexed (Figure 2D). Anterior arm is on right of intestine, posterior one is on left of intestine; anterior branch (extending 619–930 µm) is longer than the posterior one (long 586–845 µm) in most cases. Each ovary anteriorly contains a well-developed central rachis surrounded by a layer of germ cells in the reflexed part, which was referred to as “germigen” in *C. elegans* [24]. Elongated oocytes are situated proximally in a single row. Both distal parts of the uteri (40–80 µm×26–44 µm) are filled with sperms (4–7 µm in diam.) and look conspicuous in the gonad (Figure 2D). Spermatheca formed by the oviduct is often invisible, but it becomes visible when sperms are present (Figure 2E). Vulva is positioned in the mid-body region (V = 48–51) with vulval lips slightly protruding. Uteri usually carry three to ten embryos depending on the age of the female. Tails vary in length from 201 to 264 µm, tapering to a filiform terminus. Phasmids seem being located posterior to the anus about two times of the anal body width, but they have not yet been clearly observed in this study.

#### Male

 Males are common in the cultures. Testis is single-armed with anteriorly reflexed. Spermatogonia are arranged in three to four rows in the reflexed part; well-developed spermatocytes are situated proximally in two to four rows; then mature spermatids are in multiple rows in the remainder of the testis. Caudal region is enveloped by a closed bursa, which is supported by nine pairs of genital papillae (Figure 3E, F, G & H). Spicules are paired narrow shafts, separated, slightly arched ventrally at 25% from the anterior end then smoothly tapered to a pointed distal terminus. Gubernaculum is 73–86% the length of the spicule, long and thin, with distal part protruding outside of the cloaca and carrying two lateral ears as well as an indistinct forked-terminus (Figure 3F & G). Bursa looks heart-shaped in ventral view (Figure 2H), anteriorly closed with serrated edge (Figure 3E, F, & G). The nine pairs of genital papillae (GP) or rays are arranged as (2/1+3+3) (see Figure 2I; 3E, G & H) or (v1, v2)/v3 (v4, ad, v5)(pd, v6, v7) [25]. The openings of GP1 ( =  v1), GP5 ( =  ad) and GP7 ( =  pd) are to the dorsal surface of the velum being typical papilliform sensilla (Figure 3F & G); others (v2 to v7) to the ventral surface. No free sensillum tip is visible on GP6. GP2 and GP6 thicken at the base. GP2, GP4, GP5 and GP7 do not extend to near the velum edge. Precloacal lip bears a distinct projecting hook (long 4–6 µm), having three points instead of only one (Figure 3E, F, G). Phasmids are indistinct in the male. Tail is relatively short and terminus-pointed.

### Type host and locality

The type specimens of *Caenorhabditis sinica* sp. n. were collected from strain ZZY0401 established with individuals isolated from a rotten fruit in Luofu Mountain of Huizhou City, Guangdong Province, China (23°N, 114°E).

The other four isolates of *C. sinica* sp. n. in this survey were also obtained within Guangdong Province. Strain ZZY0402 was established with individuals from rotten leaves in the town of Dacheng in Xinyi County, Maoming City (22°N, 111°E). Strain ZZY0403 was established from a snail in the village of Gankeng, Genzi Town, Gaozhou County, Maoming City (22°N, 111°E). Strain ZZY0413 was established from a rotten plant tissue in Sanlingshan Forest Park of Zhanjiang City (21°N, 110°E). Strain ZZY0414 was established from rotten leaves in Luofu Mountain of Huizhou City (23°N, 114°E).

### Type materials

Twelve paratype slides (No. T-6269p to T-6280p) containing females, males and juveniles of *Caenorhabditis sinica* sp. n. strain ZZY0401 are deposited in the USDA Nematode Collection, Beltsville, MD, USA. Additional type slides are deposited in School of Life Sciences, Tsinghua University, Beijing, China. Mass-fixed specimens in 3% formalin are available at Department of Biology, Hong Kong Baptist University, Hong Kong, China.

Living worms of the type isolate ZZY0401 and other isolates in this survey (ZZY0402, ZZY0403, ZZY0413 and ZZY0414) are cryogenically preserved and deposited at the Department of Biology, Hong Kong Baptist University, Hong Kong, China. They all will be publically available at the CGC (*Caenorhabditis* Genetics Center) upon publication of the description.

### Diagnosis and relationships

*Caenorhabditis sinica* sp. n. is characterized by the following features, including a relatively large female body (long 1.1–2.0 mm) and a relatively small male body (long 0.7–1.2 mm), gonochoristic reproductive mode, presence of three ridges flanked by two incisures in the lateral field, a tube-like buccal capsule (16–24 µm×2–5 µm) with conspicuous cheilorhabdion, six small labial sensilla present, four cephalic sensilla displayed by males only, a typical rhabditid pharynx bearing a prominent cardia, a well-developed didelphic-amphidelphic female reproductive system with a mid-body vulva (V = 48–51) and two large distal parts of uteri filled with sperms, a pair of spicule shafts (separated), a long and thin gubernaculum with distal part protruding outside of the cloaca, a closed male caudal bursa supported by nine pairs of genital papillae with GP1, GP5, and GP7 terminated on the dorsal side of the velum, a uniquely shaped male precloacal hook structure having three points, and a long filiform female tail.

*Caenorhabditis sinica* sp. n. belongs to the *Elegans* group in the genus with the morphology of stoma, pharynx, as well as the male caudal bursa and genital papillae [26]. The new species possesses a uniquely shaped, three-pointd hook structure on the male precloacal lip (see Figure 3 E, F &G), which is morphologically identical to the reference strain JU727 and can differentiate this species from all other *Caenorhabditis* species. *C. sinica* sp. n. differs from the wild-type *C. elegans* and *C. briggsae* not only in the reproductive mode (gonochoristic vs. androdioecious), but also in the morphology of the two distal parts of uteri in the female/hermaphroditic gonad (filled with sperms, large and conspicuous vs. small and indistinct, see Fig 2E & J). *C. sinica* sp. n. differs from *C. brenneri* and *C. remanei* in the shape of precloacal hook (three points vs. only one point) [26]. *C. sinica* sp. n. can be also distinguished from *C. brenneri* by a longer spicule length (38.7–46 µm vs. 27–38 µm) and a relatively smaller “a” value (15.5–20 vs. 17.5–21.9 in the female and 15.8–23 vs. 20.4–29.8 in the male) [26], which indicate that *C. brenneri* is a little more slender than *C. sinica* sp. n. based on the measurements yet performed in nonparallel conditions. *C. sinica* sp. n. can be differentiated from *C. formosana* by the morphology of male genital papillae (GP4 and GP5 of *C. formosana* basally fused) [27]. *C. sinica* sp. n. differs from *C. oncomelaniae* in the morphology of female gonad (the anterior arm of female gonad is always shorter than the posterior one for *C. oncomelaniae*, yet the opposite is the case for *C. sinica* sp. n.) [27].

### Molecular profiles

For molecular analysis, three fragments of ribosomal DNA of the type isolate ZZY0401 of *Caenorhabditis sinica* sp. n. were sequenced. The 686-bp fragment including the complete sequence of internal transcribed spacer 2 (ITS2) (KF732842) matches well with those of JU727 (JN636142) and SB378 (JN636091). Sequence alignment between *C. sinica* sp. n. and strain JU727 yielded 686 total aligned nucleotides with only one nucleotide difference. Sequence alignment between *C. sinica* sp. n. and strain SB378 yielded 638 total aligned nucleotides with two nucleotide differences. However, this fragment only showed modest alignment with that of any other closely related *Caenorhabdits* species available in GenBank, with the highest identity is only 82% from *C. briggsae* (JN636061 and JN636106). The 905-bp LSU D2/D3 sequence of *C. sinica* sp. n. (KF732844) is totally identical to that of JU727 (JN636142). A BLASTN search of the 1618-bp partial SSU sequence of *C. sinica* sp. n. (KF732843) produced a closest match again to the JU727 (EU196000), in which the sequence alignment yielded 1618 total aligned nucleotides with one substitution and one nucleotide insertion.

Only one fragment of ribosomal DNA including the complete sequence of ITS2 was sequenced for other isolates (ZZY0402, ZZY0403, ZZY0413, ZZY0414). The rRNA sequences of 684-bp of ZZY0402 (KM068129), 690-bp of ZZY0403 (KM068130), as well as 686-bp of ZZY0413 (KM068131) are identical to that of JU727 (JN636142), but differences of four nucleotides were detected in the alignment between the 686-bp rRNA sequence of ZZY0414 (KM068132) and that of JU727. The ITS2 barcode sequences along with the rRNA sequences derived from our isolates supported that JU727, SB378 and our five isolates (ZZY0401, ZZY0402, ZZY0403, ZZY0413, ZZY0414) are different strains of *Caenorhabditis sinica* sp. n.

### Hybrid viability between our five isolates and the reference strains (JU1201 and JU727)

**Figure 4:**
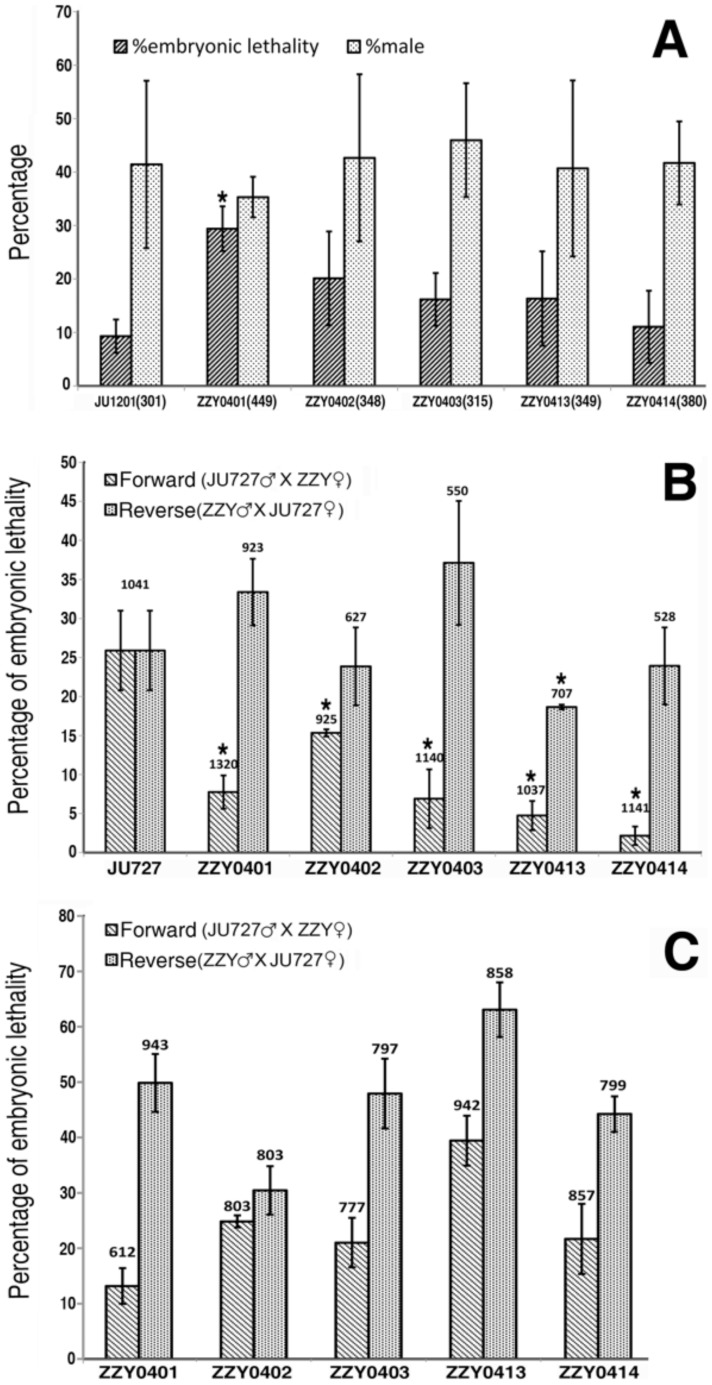
Hybrid viability between our five wild isolates and *Caenorhabditis sinica* sp. n. reference strains. A: percentage of male and embryonic lethality for *C. sinica* sp. n. (JU1201) itself and its F1 crossing progeny with our five wild isolates. Shown are data derived from one crossing direction, i.e., crossing between JU1201 males and ZZY females. Significant difference (p<0.05, Student's t test) between JU1201 and the crossing progeny is indicated with “*”. Numbers of adult progeny counted are indicated in the bottom (in parenthesis). Error bars denote standard deviations. B and C: percentage of embryonic lethality of F1 and F2 hybrid progeny between *C. sinica* sp. n. (JU727) and the five wild isolates respectively. Crossings were performed in both forward and reverse directions as indicated (See Materials and Methods for details). F1 hybrid males and females derived from the crossing in either direction were mated and F2 embryonic lethality was scored for their progeny. Significant difference (p<0.05, Student's t test) in the percentage of embryonic lethality between JU727 and the crossing progeny is marked with “*”. Numbers of total embryos counted are indicated above each bar.

Mating tests show that all strains in this study (ZZY0401, ZZY0402, ZZY0403, ZZY0413 and ZZY0414) are cross-fertile with two extant strains of *Caenorhabditis sinica* sp. n., i.e., JU1201 and JU727 (Figure 4), which were previously identified as *C.* sp. 5 [1], [14]. Both percentage of male and embryonic lethality were scored for the crossing progeny with JU1201, whilst only embryonic lethality was scored for the crossing progeny with JU727. The percentage of male is a bit lower than the expected 50% for all the crossings and the control (crossing within JU1201) (Figure 4A), which might be the result of vigorous males crawling up the sides of plates and not being counted. Surprisingly, the *C. sinica* sp. n. control strains demonstrate substantial difference in fitness in terms of embryonic lethality. Less than 10% embryonic lethality was observed for JU1201 while over 25% was observed for JU727 in their inbreeding progeny (Figure 4A & B). Intriguingly, phenotypic characterization of hybrid progeny between JU727 and the five isolates revealed crossing-direction dependent embryonic lethality. Embryo viability was significantly improved in all of the five crossing progeny with JU727 males compared with inbreeding JU727 progeny. Similar improvement was only observed for ZZY0413 if the crossing was conducted in the opposite direction. The high embryonic lethality observed for JU727 is likely produced by inbreeding depression while the improvement in embryonic viability possibly reflects inhibition of the inbreeding depression by the outcrossing with the five isolates, supporting that all the crossing parents belong to the same species. To further confirm the conspecificity between JU727 and the five isolates, we performed crossing in both directions between the surviving F1 males and F1 females because some speciation events were shown to produce significant breakdown only in F2 hybrid progeny [28]. It was interesting that the breakdown in F2 hybrid progeny was indeed the case for the crossings (Figure 4C), but the level of the breakdown appears not to be sufficient to define the crossing parents into two different species. It is worth noting that the crossing-direction dependent bias in embryonic lethality still holds in F2 generation albeit at a reduced differentiation.

## Discussion

*Caenorhabditis sinica* sp. n. can be easily differentiated from the wild strains of *C. elegans* and *C. briggsae* by the presence of male having roughly equal number to the female, and the detailed morphology of the female/hermaphroditic gonad. However, the new species is difficult to be distinguished morphologically from other gonochoristic *Caenorhabditis* spp. within the *Elegans* group, e.g. *C. brenneri* and *C. remanei*. Kiontke et al. [13] defined the evolutionary phenotypic characters at male tails within this group, where a heart-shaped fan of male tail always co-occurred with a hook-shaped precloacal lip. In this study, we used the characteristic precloacal hook of *C. sinica* sp. n. to compare its morphological similarity with the reference strain JU727 for distinguishing this species from all other *Caenorhabditis* species.

Consistent with previous report [13], the ITS2 sequence turned out to be a useful and sufficient barcode for distinguishing *Caenorhabditis* spp. from one another. Unlike the conservative sequences of LSU (28S) and SSU (18S) rRNA genes, ITS2 sequences are variable between the closely related *Caenorhabditis* species. Sequence alignments of ITS2 between *C. sinica* sp. n. and its phylogenetically closest species, *C. briggsae*, as well as *C. nigoni*, a species was previously referred to as *C.* sp. 9 but recently named formally [29], revealed that the DNA sequence similarities were only 82% and 81% respectively. In contrast, the identity of all ITS2 sequences within *C. sinica* sp. n. including all the new isolates (ZZY0401, ZZY0402, ZZY0403, ZZY0413, ZZY0414) and the extant isolates (e.g. JU727 and SB378) is more than 99%, providing molecular evidence that all these isolates belong to the same species.

The high F1 embryonic mortality in JU727 and breakdown of the F2 hybrid progeny between JU727 and our five isolates (Figure 4 B&C) suggest partial reproductive barriers between some genotypes of *Caenorhabditis sinica* sp. n. However, the ITS2 sequences and morphological data of the strain ZZY0401 as well as the incomplete F2 breakdown data support the taxonomic position of our five isolates is identical to that of the reference strain JU727. The F1 crossing progeny between JU727 and our five isolates are ready to mate and their F2 progeny does show breakdown to certain extent, but far from being complete. The highest F2 breakdown was observed between ZZY0413 and JU727, which was measured as approximately 65% embryonic lethality in a crossing-direction manner (Figure 4C). The F2 breakdown with ZZY0401, the strain used for morphological description, is around 50% in reverse direction and 13% in forward direction. Future work is needed to determine the crossing viability between these isolates. All five isolates demonstrated similar crossing-direction dependent bias in embryonic lethality, which is interesting. Whether the asymmetry represents nuclear-cytoplasm hybrid incompatibility remains unclear. It is worth noting that 65% F2 breakdown between JU727 and ZZY0413 suggested at least some subpopulations of *C. sinica* sp. n. are present genetically based on their clustered localities. This does not agree well with the results based on DNA sequence divergence of X-linked coding genes, which predicted that *C. sinica* sp. n. would not exhibit differentiated subpopulation [14]. The discrepancy highlights the importance of mating test in defining the population structure of a given species. Widespread sex-biased hybrid lethality in F1 and substantial F2 breakdown along with its extreme molecular diversity make *C. sinica* sp. n. an attractive model for study of population and speciation genetics in the future.
